# Protocol for a winter sentinel surveillance program of notifiable respiratory viruses in Queensland

**DOI:** 10.1371/journal.pone.0277895

**Published:** 2022-11-28

**Authors:** Shamila Ginige, Elise Firman, Yee Sum Li, Yudish Soonarane, Nicolas Smoll, Fiona May, Ian Hunter, Brielle Pery, Bonnie Macfarlane, Tracy Bladen, Terresa Allen, Melinda Lennon, Jacina Walker, Vicki Slinko, Mark Stickley, Gulam Khandaker, Satyamurthy Anuradha, Andre Wattiaux

**Affiliations:** 1 Gold Coast Public Health Unit, Gold Coast Hospital and Health Service, Cararra, Australia; 2 Metro South Public Health Unit, Metro South Hospital and Health and Service, Brisbane, Australia; 3 Central Queensland Public Health Unit, Central Queensland Hospital and Health Service, Rockhampton, Australia; 4 School of Public Health, University of Queensland, Brisbane, Queensland, Australia; University of Helsinki: Helsingin Yliopisto, FINLAND

## Abstract

**Background:**

With the reduction in access to polymerase chain reaction (PCR) testing and changes in testing guidelines in Australia, a reduced number of people are seeking testing for coronavirus disease (COVID-19), limiting the opportunity to monitor disease transmission. Knowledge of community transmission of COVID-19 and other respiratory viruses is essential to better predict subsequent surges in cases during the pandemic to alert health services, protect vulnerable populations and enhance public health measures. We describe a methodology for a testing-based sentinel surveillance program to monitor disease in the community for early signal detection of SARS-CoV-2 and other respiratory viruses.

**Methods/design:**

A longitudinal active testing-based sentinel surveillance program for respiratory viruses (including SARS-CoV-2, influenza A, influenza B and Respiratory Syncytial Virus) will be implemented in some regions of Queensland. Adults will be eligible for enrolment if they are part of specific community groups at increased risk of exposure and have not had a COVID-19 infection in the last 13 weeks. Recruitment via workplaces will occur in-person, via email and through online advertisement. Asymptomatic participants will be tested via PCR for SARS-CoV-2 infection by weekly self-collected nasal swabs. In addition, symptomatic participants will be asked to seek SARS-CoV-2 and additional respiratory virus PCR testing at nominated COVID-19 testing sites. SARS-CoV-2 and respiratory virus prevalence data will be analysed weekly and at the end of the study period.

**Discussion:**

Once implemented, this surveillance program will determine the weekly prevalence of COVID-19 and other respiratory viruses in the broader community by testing a representative sample of adults, with an aim to detect early changes in the baseline positivity rate. This information is essential to define the epidemiology of SARS-CoV-2 in the community in near-real time to inform public health control measures and prepare health services and other stakeholders for a rise in service demand.

## Introduction

By 29 August 2022, SARS-CoV-2 had infected more than 596 million people, caused over 6 million deaths worldwide [1], and indirectly impacted many more, cementing its place as a significant public health issue. In Australia, the first case was detected in January 2020 and since then has infected more than 8.6 million people and led to over ten thousand deaths [2].

Reduced public health measures and waning vaccine immunity against infection increases the spread of SARS-CoV-2 in the community. Initial public health and social measures that successfully controlled the rapidly rising numbers of SARS-CoV-2 cases have been gradually removed. Although COVID-19 vaccination coverage in Australia is high, with over 95% of adults fully vaccinated [3], studies have shown waning immunity with time since immunisation [4]. This, along with a doubling time for SARS-CoV-2 of three days [5], highlights the need for early identification and investigation to reduce spread of infection.

Sentinel surveillance programs have been used for early detection of other respiratory viral illnesses, including influenza and Respiratory Syncytial Virus (RSV). They have provided reliable and timely data, sometimes pre-dating the increase in positive laboratory samples by two weeks [6–10]. However, disease characteristics of SARS-CoV-2 make early detection difficult, including a large proportion of asymptomatic and pauci-symptomatic presentations, and an increased lag time to positive laboratory diagnosis [11, 12]. Cryptic transmission phases have been described in previous waves [13, 14], and with a pre-symptomatic infectious period of one to three days [15], the utility of traditional syndromic surveillance approaches is reduced. Therefore, a testing-based surveillance system is better for opportunistic early detection of SARS-CoV-2 and other respiratory viral pathogens. A literature review found alternate methods to describe SARS-CoV-2 epidemiology [16–18], but found no current testing-based surveillance systems in Australia to monitor the status of disease transmission in the community. The authors believe this project will address this gap and be a novel surveillance program for early wave detection in Australia.

Concerningly, the incidence of other winter respiratory viruses in 2022 could be much higher than in previous years due to waning immunity and reduced circulation of these viruses in the last two years. Given the easing of public health restrictions and reduced number of people seeking testing for SARS-CoV-2, a diagnostic sentinel surveillance program for respiratory viruses, particularly in the lead up to winter, is important to predict another wave of COVID-19, influenza, and other respiratory pathogens. This will also allow vulnerable settings (for example, residential aged care facilities) to take necessary measures, including increased infection control and public health measures during sustained community transmission, to protect their populations and reduce the risk of outbreaks.

Targeting a selected population with an increased SARS-CoV-2 infection risk early in an outbreak may allow earlier detection of another ‘wave’. For example, the first surge in case numbers in Queensland occurred in December 2021 after the state borders were opened, with the 18–39-year-old age group among the first to see case numbers rise. Several international analyses found the early warning population included those who work in occupations unable to be performed from home and in environments where physical distancing and effective control measures are challenging [19–21].

## Materials and methods

### Aim

This project will implement a testing-based sentinel surveillance program for SARS-CoV-2, influenza, and RSV in local areas over winter. Monitoring trends may help further characterise the epidemiology of SARS-CoV-2. In addition, it may detect signals of increasing case numbers for respiratory diseases, consistent with a new surge that may impact hospital admissions. This would also help establish whether self-collected nasal PCR swabs by untrained individuals have a role in testing-based sentinel surveillance.

### Design

Longitudinal active testing-based sentinel surveillance program for SARS-CoV-2 with additional influenza and RSV testing for symptomatic participants only.

### Setting

This program will be implemented in collaboration with several Public Health Units (PHUs) in Queensland, Australia. Each PHU will recruit participants from a range of age groups and occupational backgrounds to capture those who may be most at risk of infection.

### Sample size

A time-space sampling strategy will be used focussing on location-based populations that may be at risk of contracting a respiratory virus earlier. This probability-based strategy will allow recruitment of members from target populations that congregate at specific locations and times [22–24]. Respondent-driven sampling will be used if it is recognised that project participants can refer other potential participants with whom they have an established relationship.

The sample size will be calculated to detect a change of 1% from a hypothesized percent positivity rate based on a 95% confidence interval for each population.

### Inclusion criteria

1. ≥18 years of age
2. Part of a select community group within Queensland likely to be at increased risk of exposure, for example hairdressers, healthcare workers, police officers, paramedics, supermarket/retail workers, hospitality staff, theme park employees, university and vocational students and schoolteachers.

### Exclusion criteria

1. <18 years of age
2. Inability to give informed consent
3. Diagnosis of SARS-CoV-2 infection within previous 90 days. This period was chosen to be reasonably confident that a new detection of SARS-CoV-2 reflects an actual new infection [25], rather than viral shedding. This exclusion criterion may be changed to align with local public health guidelines that reflect viral characteristics of the dominant variant in the area at that time.

### Program procedures

#### Recruitment and withdrawal

Community groups whose employees/members may be at a higher risk of SARS-CoV-2 infection early in a wave will be identified and approached to participate in the program. This may be through in-person contact, by email or online advertisement. Information about the program will be provided to promote the program within the community group with a link to an electronic registration form. Participant details and consent will be collected as part of the registration form (Table 1).

**Table 1:** Data collected from participants at recruitment.

Demographic data	First name
	Surname
	Date of birth
	Gender
	Occupation
	Place of occupation
	Residential address
	Indigenous status
	Mobile number
	Email address
COVID-19	Vaccinated: Y/N
	Vaccine name/s
	Vaccinated date/s:
	Dose 1. DD/MM/YYYY
	Dose 2. DD/MM/YYYY
	Dose 3. DD/MM/YYYY
	Additional doses?
	Previous positive COVID test date/s and type of test (RAT or PCR)
Other pathogens	2022 Influenza vaccine: Y/N
	Vaccine name
	Vaccine date: DD/MM/YYYY
	Previous positive influenza/RSV test date/s in 2022

RAT: Rapid Antigen Test; PCR: Polymerase Chain Reaction; RSV: Respiratory Syncytial Virus

Participant numbers are expected to decline throughout the program due to withdrawal, loss to follow-up and participant exclusion with diagnosis of SARS-CoV-2 infection (primary endpoint). Therefore, rolling recruitment is expected to be required to retain appropriate study power to detect statistically significant changes in case positivity rate.

Participants can withdraw from the program at any time by contacting the surveillance program team directly or completing an online survey (the link will be provided in the welcome pack information). Unless specifically requested otherwise, data collected before a withdrawal will continue to be used in the analysis. After the program, arrangements will be made to gather participants’ feedback.

#### Testing

Participant testing will be based on the presence of symptoms and categorised into two streams—asymptomatic participants and symptomatic participants.

*Asymptomatic participants*. Participants will be provided with a welcome pack that includes pre-filled pathology request forms specifically coded to identify inclusion in the surveillance program, pre-labelled nasal swabs for testing use and an instruction sheet detailing the nasal swab self-collection method. Participants will self-collect a nasal swab unobserved once per week for the duration of the program and deposit their swab in a designated collection box at their workplace or other designated site on a pre-determined day. A PHU employee will collect and transport these swabs to the pathology laboratory for SARS-CoV-2 PCR testing. A reminder text message and email will be sent to participants weekly to maximise ongoing participation in the program.

*Symptomatic participants*. Participants who become symptomatic will be asked to attend a COVID-19 testing centre with a ‘symptomatic’ pathology form. This pathology form will be pre-filled with participant details requesting a specific respiratory panel (for influenza A, influenza B and RSV) in addition to SARS-CoV-2. It will be specifically coded to identify inclusion in the surveillance program for data download purposes. Additionally, detailed information on seeking urgent and appropriate medical care for acute symptoms will be provided.

#### Endpoint for participants

Participants who have SARS-CoV-2 detected will be considered to have completed the program and will not be required to provide any further swabs. Those with another respiratory pathogen detected will remain in the program until SARS-CoV-2 is detected on their specimen, or until the program concludes; whichever is earlier.

### Outcomes measured

#### Primary outcome

Changes in the proportion of cases of COVID-19 and other pathogens in participants compared to previous weeks, to detect a significant rise in cases.

#### Secondary outcome

Weekly prevalence of asymptomatic and symptomatic COVID-19 infections amongst participants selected from specific community groups.

Weekly prevalence of symptomatic respiratory virus infections (influenza A, influenza B and RSV) amongst participants selected from specific community groups.

### Data collection

#### Data variables required

Tables 1 and 2.

**Table 2:** Data collected weekly from asymptomatic and symptomatic participants who are tested.

Asymptomatic participant	SARS-CoV-2 test result
Symptomatic participant	SARS-CoV-2 test result
	Influenza A and B test results
	RSV test result

RSV: Respiratory Syncytial Virus

*Planned data analyses*. Missing data will be coded as such. Data will be analysed weekly (Table 2) to assess change in positivity in the cohort for early detection of a potential COVID-19 ‘wave’ and increases in other respiratory pathogens. Results will be presented as descriptive statistics. Where required, means, differences in means or medians, proportions and their corresponding 95% confidence intervals will be presented.

With the variability in SARS-CoV-2 case numbers over the last two and a half years, the emergence of new dominant variants and changes to public health measures, it has been difficult to establish baseline rates and compare them with historical tests. Consequently, data from the first three weeks of collection will be used to establish a baseline for the current public health measures, with subsequent weeks to be compared for early detection of increasing cases. A threshold value for early signal detection can be established by adapting the alert threshold from Global Epidemiological Surveillance Standards for Influenza methods; 1.645 standard deviations above the mean for each week, defines the 90% confidence interval of the mean [26].

### Ethical considerations

An ethics exemption was granted from the Gold Coast Hospital and Health Service Human Research Ethics Committee (EX/2022/QGC/84722) as the program will be a quality activity and part of routine public health surveillance.

### Status and timeline of the study

Recruitment of participants will commence in March with weekly testing beginning in April. The Sentinel Surveillance Program will continue for six months from April until September (cooler months in the southern hemisphere). Recruitment will be ongoing until September. New data will be analysed weekly for primary and secondary outcomes. Data analysis will be conducted in October after the end of the program.

## Discussion

In this paper, we describe an active testing-based sentinel surveillance program to detect and monitor respiratory viruses, including SARS-CoV-2, in a selected early warning cohort of community groups within a local area. The program aims to report the weekly prevalence to monitor trends in respiratory viruses over winter with the intent of early signal detection of SARS-CoV-2 and specific respiratory viruses (influenza A, influenza B and RSV). This information will help better understand the epidemiology of SARS-CoV-2. In addition, it may be used to guide public health action and prepare health services for a possible increase in service demand because of increased infections in the community. The surveillance program will be concurrently run over several geographical areas by different PHUs working closely together to ensure consistency in program implementation.

### Strengths

The project is a novel active testing-based sentinel surveillance program supporting surveillance objectives of PHUs at the forefront of pandemic and outbreak responses. It will generate accurate local information on disease transmission and epidemiology to allow more measured public health responses with early communication to healthcare systems for improved planning and preparedness. This will help achieve essential care continuity, reducing disruption and improving health outcomes for the population. An evidence-based approach also reassures the community that the public health response remains proportional to risk. Government officials and decision-makers often seek assurance that reported case numbers reflect the true level of disease in the community. However, this cannot be reliably established from the current passive surveillance systems but should be provided through this program.

The data generated by the program could contribute to national influenza sentinel surveillance programs, providing additional data at minimal cost to help capture the disease burden of RSV–important as RSV has recently been made notifiable in Queensland.

### Limitations

There are several limitations to this study design. First, the program’s design hinges on a large sample size to provide statistically significant data. Workplaces are the target sites, but positive results of SARS-CoV-2 could impact the workforce. This may limit the number of organisations and individuals interested in participating leading to selection bias in the sample population. This program was not intended to be a surveillance system to calculate the burden of disease in the whole population but to detect an early change in a baseline rate of case numbers. People under 18 years were excluded from the study due to ethical considerations around the invasive nature of the procedure. Their exclusion should not impact on the objectives of the program.

Testing of symptomatic participants will be crucial but as they are unable to enter their workplace with symptoms, facilitating testing is a challenge. The designated COVID-19 testing centres are an option for this cohort but with limited centres, this may be a barrier to testing. Participants may seek testing outside of this program, for example rapid antigen tests or private pathology laboratories.

The gold standard investigation for SARS-CoV-2 infection is clinician-collected nasopharyngeal swab for PCR. However, utilising this method could substantially reduce participation due to the impost of having to attend a testing centre weekly. Given the target sample size, the logistics of clinicians attending workplaces weekly for nasopharyngeal swab collection from participants is difficult. Therefore, self-collected nasal swabs will be used to still achieve reasonable test reliability with few barriers to participation; compared to rapid antigen tests or saliva tests.

Studies have looked at the reliability of self-collected swabs for SARS-CoV-2 compared to those collected by healthcare workers. One study found that self-collected nasal and throat swabs were a reliable alternative with concordant results and little difference in results for E-gene and N-gene [27]. Another found sensitivities of self-collected oropharyngeal and mid-turbinate swabs were inferior but could still be useful in an appropriate clinical setting [28].

Compared to clinician-collected specimens delivered to a testing laboratory in a timely manner, our methods will result in specimens being transported and stored in uncontrolled environments for possibly extended periods. A study showed that dry swab samples for SARS-CoV-2 are stable at room temperature for 24 hours [29], but degradation of sample is possible if there are delays of up to 48 hours between collection and testing. To minimise delays, participants will be instructed to self-collect their swab on the morning of the designated collection day.

Lastly, testing for all common respiratory viruses on all participants (whether asymptomatic or symptomatic), would provide a much richer data set and contribute to better understanding the clinical significance of asymptomatic infections. However, this protocol has been developed to maximise both utility and cost-effectiveness, so testing regimes are streamlined into two pathways depending on whether the participant has symptoms. This is also to protect laboratory capacity given the anticipated high rates of community testing for respiratory viruses as winter approaches.

### Dissemination plans

A weekly summary of de-identified prevalence results with trends will be distributed to all participants during the program. Health service executives will have access to these weekly summaries so that local hospitals can receive timely notification of any signal event through the hospital communication team. Executives from other services involved (e.g., police and universities) will also receive the regular summaries on trends so they can consider tailoring their services accordingly. An overall summary of findings will be presented at the end of the program with a view to publish in relevant peer-review journals and presented at national and international conferences.

### Amendments, and criteria for closure

Throughout the program, team members will have regular meetings to assess the program and discuss amendments to the protocol as required. Any modifications to the program, such as changes in eligibility criteria or testing schedules, will be communicated to participants.

The program will be terminated in September 2022 after the end of winter. The program duration may be shortened if a new SARS-CoV-2 wave is detected, given the limited continuation utility after the program achieves its primary purpose. Conversely, the program may be extended if ongoing surveillance is required, for example emergence of a new variant.

A formal evaluation will occur after the program to identify strengths and opportunities for improvement. This will enable future programs to adapt procedures towards more efficient and effective practice.
